# Epigenetic age acceleration in young adults with congenital heart disease

**DOI:** 10.1186/s13148-026-02049-5

**Published:** 2026-02-15

**Authors:** Parag N. Jain, Beryl C. Zhuang, Joanne Whitehead, Julia L. MacIsaac, Kristy Dever, Mallory Gahm, Peter Ermis, Thomas W. McDade, Michael S. Kobor, Paul A. Checchia

**Affiliations:** 1 Division of Cardiology, Heart Center, Children’s Health, Department of Pediatrics, UT Southwestern Medical Center, 1935 Medical District Drive, Dallas, TX 75235 USA; 2 Edwin S.H. Leong Centre for Healthy Aging, University of British Columbia, Vancouver, BC V6T 1Z4 Canada; 3 Centre for Molecular Medicine and Therapeutics and Department of Medical Genetics, University of British Columbia, Vancouver, BC V5Z 4H4 Canada; 4 BC Children’s Hospital Research Institute, 950 West 28Th Avenue, Vancouver, BC V5Z 4H4 Canada; 5 Texas Children’s Hospital and Baylor College of Medicine, Houston, TX USA; 6 Department of Anthropology, Northwestern University, Evanston, IL USA; 7 Institute for Policy Research, Northwestern University, Evanston, IL USA

**Keywords:** Congenital heart disease, Fontan circulation, Epigenetic clocks, Epigenetic age acceleration

## Abstract

**Background:**

Adults with congenital heart disease (ACHD) having undergone palliative surgery experience chronic stress due to altered physiology and repeated surgical interventions since infancy.

**Objectives:**

To investigate whether ACHD, who had experienced chronic physiological stress from their underlying condition and early-life cardiac surgeries, was associated with epigenetic age acceleration (EAA) and other DNA methylation (DNAm)-based biomarkers, and to assess the potential contribution of derived inflammatory markers to EAA.

**Methods:**

A case–control study comparing ACHD patients and healthy adults. Whole blood DNAm profile was used to estimate DNAm-based blood cell type proportions, multiple epigenetic age measures, and interleukin-6 (IL-6) and C-reactive protein (CRP) scores. Two ACHD subgroups were recruited: one with multiple palliative surgeries since birth (Fontan group, *n* = 13), and another with a single corrective surgery as an infant (SS group, *n* = 5). Healthy controls (*n* = 20) had no chronic medical conditions. EAA was calculated using four epigenetic clocks (Horvath, Hannum, GrimAge, PhenoAge) and the pace of aging (DunedinPACE). Comparisons were made across groups using robust linear regression models, adjusting for age, sex, self-reported ethnicity, and estimated cell type proportions. Associations between DNAm-based IL-6 and CRP scores and surgery group were tested, and their potential contribution to differences in EAA was evaluated.

**Results:**

Participants were 20–30 years (25.6 ± 2.7 years), predominantly non-Hispanic white. After controlling for age/sex/ethnicity/immune-cell-type proportions, the Fontan group had significantly higher GrimAge (Cohen’s f = 0.90, *p* < 0.001) and PhenoAge (Cohen’s f = 0.82, *p* < 0.001) and higher DunedinPACE (Cohen’s f = 0.69, *p* = 0.01). The Fontan group also had statistically higher predicted IL-6 (Cohen’s f = 0.84, *p* < 0.001) and CRP scores (Cohen’s f = 0.62, *p* < 0.001).

**Conclusions:**

Young ACHD patients who undergo multiple childhood surgeries for Fontan palliation were associated with accelerated aging. These changes could reflect the long-term effects of underlying CHD condition, early-life physiological stress and other factors, potentially involving inflammatory pathways. Further research is needed to identify and validate the key factors contributing to EAA in this population and to clarify the role of chronic stress and physiological alterations over time.

**Supplementary Information:**

The online version contains supplementary material available at 10.1186/s13148-026-02049-5.

## Background/Introduction

Congenital heart disease (CHD) is the most common birth defect in the US, affecting 1% of live births [1]. Advances in care have improved survival for adults with congenital heart disease (ACHD) [2, 3]. However, ACHD patients face significant comorbidities due to imperfect hemodynamics, repeated surgeries, and chronic inflammation, with higher mortality burden in more severe cases [4–7]. ACHD patients with Fontan circulation undergo three stages of surgical palliation, starting with the Norwood procedure as neonate, Glenn procedure at 4–6 months of age, with final completion of Fontan circulation by age 2–3 years, resulting in passive systemic venous return to the heart. As a result, ACHD with Fontan circulation suffer chronic stress due to altered physiology [8] and lower physical health status [2], and increased age-related comorbidities like dementia and coronary artery disease, with early signs of cardiac aging due to excessive inflammation and oxidative stress stress [9].

DNA methylation (DNAm), which involves adding a methyl group to the 5’ position of cytosine at cytosine-guanine dinucleotides (CpGs), plays a key role in several important biological processes, including development, cell differentiation, and imprinting [10]. The accumulation of DNAm data has led to the development of DNAm-based tools, such as cell type deconvolution algorithms [11, 12], epigenetic clocks [13–17], and DNAm-based biomarker predictors [18, 19]. which are widely used in health-related epigenetic research [20]. Among all these tools and biomarkers, research and development of epigenetic clocks is a prominent and rapidly advancing field. Epigenetic clocks use DNAm patterns at specific CpGs that are highly correlated with chronological age [21–23], and can be used as biomarkers to investigate premature aging in CHD [2, 7]. Epigenetic age acceleration (EAA), is a derived measure of rate of aging [24, 25], and higher EAA has been associated with negative outcomes of various health conditions and diseases, such as cardiovascular disease [14–16, 26–28] and metabolic syndrome [17, 29].

The biological embedding of early life experience is the process by which childhood exposures can alter developmental trajectories, leading to individual variations in health and wellbeing in adulthood [30]. DNAm acts as a bridge between environment and genome, embedding experiences like trauma into biology [30], and can be detected by DNAm-based biomarkers [31]. While epigenetic aging is studied in cardiovascular disease [32, 33], it is less explored in ACHD population, especially in young adults, when early intervention is possible. We hypothesize that the early life exposure of highly invasive surgical procedures, plus the cumulative metabolic stress and systemic inflammation imposed by altered circulatory physiology, can potentially become embedded in the epigenome of young ACHD patients. This impact may be detectable by DNAm-based tools, namely cell type proportion estimations, inflammation scores and epigenetic clocks, and with strongest impact in those who underwent multiple palliative interventions including Fontan surgery in early childhood. Here, we investigated whether ACHD, who had experienced chronic physiological stress from their underlying condition and early-life cardiac surgeries, exhibit differences in DNAm-based biomarkers compared to controls. We further explore a potential contribution of inflammation in EAA.

## Materials and methods

### Study participants description

This case–control study involved ACHD patients aged 20–30 years, and healthy controls of the same age range with no past medical or surgical history (Table 1). Two subgroups of ACHD patients were recruited during their routine outpatient visits: the Fontan group included patients who underwent multiple palliative surgical interventions during infancy and early childhood with Fontan surgery being the latest intervention, resulting in passive pulmonary circulation and increased venous congestion. The second subgroup consisted of patients with a single corrective surgery as an infant (SS group). The study was approved by the Baylor College of Medicine IRB and informed consent was obtained from all participants (cases and controls) before blood sampling. After obtaining informed consent, 5 mL of peripheral whole blood was collected via venipuncture into EDTA tubes from all patients during their routine outpatient visits with an ACHD cardiologist. All samples including controls were collected following the same protocol and were immediately stored at − 80 °C until further processing.

**Table 1:** Cohort demographics

	Control	Fontan	Single surgery	*p*-value
No. patients (Total 38)	20	13	5	
Sex				0.241
Male	5	6	3	
Female	15	7	2	
Ancestry				0.96
White non-Hispanic	13	7	4	
White Hispanic	3	2	1	
African American	2	4	0	
Asian	2	0	0	
Age at sample collection: Median (IQR)	27 years (25- 28)	23 years (21–26)	25 years (23 – 28)	
Age at last Surgery: Median (IQR)	NA	3 years (2 – 4 years)	3 months (1 – 10 months)	
Surgical repair	NA	Fontan (13)	TOF (3) ASD/VSD (1) ASO (1)	

Number of patients or controls in each group, with sex, ancestry, age at sample collection and at last surgery, and type of surgical repair. TOF, Tetralogy of Fallot repair, ASD/VSD, Atrial Septal Defect/Ventricular Septal Defect; ASO, Arterial Switch Operation. The *p*-value was calculated using chi-square test

### Microarray genome-wide DNA methylation profiling and data processing

Genomic DNA was extracted from whole blood using the Qiagen DNeasy Blood and Tissue Kit according to the manufacturer’s instructions [34]. The EZ DNA Methylation Kit (Zymo Research, Irvine, CA, USA) was used to bisulfite-convert 750 ng of genomic DNA, allowing for sequence-based differentiation of unmethylated and methylated cytosine nucleotides. An input of 160 ng of converted DNA was used for whole-genome amplification and enzyme fragmentation, then hybridized to Infinium MethylationEPIC BeadChip V1 (Illumina, CA, USA), and scanned with an Illumina HiScan. Raw IDAT files capturing the signal intensities were exported for analysis in R (version 4.2.0). Sample quality control (QC) was assessed as described previously [35, 36]. Briefly, all samples passed the QC metrics including Illumina quality control metrics [37], average methylated and unmethylated intensity [38], detection *p*-value [38], beadcount [38, 39], outlier detection [39], and predicted sex and reported sex matching [38]. Signals were background-subtracted and color-corrected, followed by Beta-Mixture Quantile (BMIQ) normalization [40], then technical batch correction was performed using ComBat from the R package sva (version 3.46.0) [41]. DNA methylation levels are reported as *β* values ranging from 0 (unmethylated) to 1 (fully methylated). Background-subtracted and color-corrected DNAm *β* values were used as input for cell type proportion estimation as recommended [11], while normalized and batch-corrected DNAm *β* values were used to calculate epigenetic clocks and DNAm-based inflammation markers since normalization had been shown to improve clock performance [42].

### Cell type proportion estimation

Cell type composition is one of the key drivers of variation in DNAm profiles [43, 44]. In the absence of direct cell counts of the whole blood samples, we estimated the relative proportions of 12 blood cell types (neutrophils, eosinophils, basophils, monocytes, naïve and memory B cells, naïve and memory CD4 + and CD8 + T cells, natural killer, and T regulatory cells) from DNAm data using the IDOL method [15]. The proportional cell type estimations were adjusted by the isometric log-ratio (ilr) transformation, and then a robust principal component analysis (PCA) was applied for dimension reduction [45].The top 3 principal components (PCs) accounted for 93.1% of predicted cell type proportion variability and were thus included in subsequent statistical models.

To determine the extent of influence of surgery group on immune cell type proportions, we applied robust linear regression models (rlm) to test estimated cell type proportion differences between the control, Fontan and SS groups, while adjusting for age, biological sex, and self-reported ethnicity:

$$ \begin{gathered} Cell\;type\;proportion\sim Surgery\;Group \hfill \\ \quad + Age + Sex + Ethnicity + \varepsilon \hfill \\ \end{gathered} $$

Each estimated cell type was tested independently using the same model. To correct for multiple testing, we applied the Bonferroni correction with threshold of adjusted *p*-value < 0.05.

### Estimation of first- and second-generation epigenetic clocks and rate-based clock

Epigenetic age as measured by the Horvath pan-tissue clock [25] (Horvath), Hannum clock [46], GrimAge [47] and PhenoAge [48] were calculated using the DNA Methylation Age Calculator (https://dnamage.genetics.ucla.edu/new), while DunedinPACE was calculated using the DunedinPACE R package [49]. Additionally, DNAmPACKYRS, one of the DNAm-based biomarkers incorporated in GrimAge to estimate smoking exposure (“pack-years”), was extracted for downstream analysis. For the first-and second-generation clocks, epigenetic age acceleration (EAA) was calculated by extracting the residuals from a linear model of DNAm age regressed on chronological age for Horvath, Hannum, GrimAge and PhenoAge estimates. DunedinPACE measures rate of aging, therefore without the need for regression on chronological age.

The differences between the three groups were tested by rlm independently for the four EAA measures and DunedinPACE, all adjusted for age, biological sex, self-reported ethnicity and top three cell type PCs:

$$ \begin{gathered} EAA/DunedinPACE\sim Surgery\,Group + Age \hfill \\ \quad + Sex + Ethnicity + Cell\,Type{\text{ }}PCs{\text{ }}1 - 3 + \varepsilon \hfill \\ \end{gathered} $$

Bonferroni correction was applied. Effect size was calculated as Cohen’s f, using the effsize R package (version 0.8.1) [50].

### DNAm-based inflammation marker predictions

DNAm-based scores of inflammation biomarkers, interleukin-6 (IL-6) and C-reactive protein (CRP), positively correlate with measured plasma levels, thus providing proxy measures of chronic inflammation [18, 19]. We calculated two DNAm-based markers of inflammation: IL-6 score [18] and CRP score [19], both transformed into z-scores (standardized to mean of 0 and standard deviation of 1). Using the same statistical model as described above, DNAm IL-6 and CRP score differences between groups were tested by the following rlm, and *p*-values were Bonferroni corrected:

$$ \begin{gathered} IL - 6\,score/CRP\,score\sim Surgery\,Group + Age \hfill \\ \quad + Sex + Ethnicity + Cell\,Type\,PCs{\text{ }}1 - 3 + \varepsilon \hfill \\ \end{gathered} $$

### Contribution analysis

To test whether the inflammation biomarkers and other predicted factors influenced EAA, we performed contribution analysis, as demonstrated previously [35, 51, 52]. We calculated the percent contribution to the regression *β* coefficient of surgery groups by including contributors: (1) DNAm IL-6 score, (2) DNAm CRP score, and (3) DNAmPACK in the adjusted models, and compared these to the base model.

$$ \begin{gathered} Base\,model:EAA/DunedinPACE \hfill \\ \quad \sim Surgery\,Group + Age + Sex \hfill \\ \quad + Ethnicity + Cell\,Type\,PCs\,1 - 3 + \varepsilon \hfill \\ \end{gathered} $$

$$ \begin{gathered} Adjusted\,model:EAA/DunedinPACE \hfill \\ \quad \sim Surgery\,Group + Contributor + Age + Sex \hfill \\ \quad + Ethnicity + Cell\,Type\,PCs{\text{ }}1 - 3 + \varepsilon \hfill \\ \end{gathered} $$

Percent contribution was calculated as,

$$ \frac{\begin{gathered} base\;\beta \;coefficient\;surgery\;group \hfill \\ \quad - adjusted\;\beta \;coefficient\;surgery\;group \hfill \\ \end{gathered} }{{base\;\beta \;coefficient\;surgery\;group}} \times 100 $$

## Results

We enrolled 13 ACHD patients in the Fontan group, 5 ACHD patients in SS group, and 20 healthy controls. The control group was predominantly White females. The Fontan and SS groups had almost equal male to female patients, and had predominantly White patients of non-Hispanic descent. Median ages at sampling were 27 years for controls, 23 years for the Fontan group, and 25 years for the SS group. The median age for the last surgery was 3 years for Fontan and 3 months for SS patients. Cohort demographics are summarized in Table 1, with cardiac anatomy and surgical details in Table S1. A power calculation accounting for the unequal sample sizes between groups (*n* = 13 for the Fontan group and *n* = 20 for controls) was performed after sample collection, as the sample size was not determined a priori using power analysis. We estimated the statistical power to detect small (Cohen’s d = 0.2), medium (d = 0.5), and large (d = 0.8) effect sizes using a two-tailed test with an alpha level of 0.05. The estimated power was 8%, 27%, and 59% for small, medium and large effect sizes, respectively, and a minimum sample size of *n* = 15 per group would be required to achieve 80% power to detect a large effect size.

### Fontan group had higher GrimAge, PhenoAge EAA and DunedinPACE

We tested the correlation of epigenetic ages from four clocks with chronological age, expecting a high correlation. GrimAge and Horvath age estimates were moderately positively correlated with chronological age (*r*(36) = 0.57, *p* < 0.001; *r*(36) = 0.49, *p* = 0.002), while Hannum had the lowest correlation and highest mean absolute error (17.96 years) of the four clocks (*r*(36) = 0.05, *p* = 0.76) (Figure S1, Table S2).

**Fig. 1:**
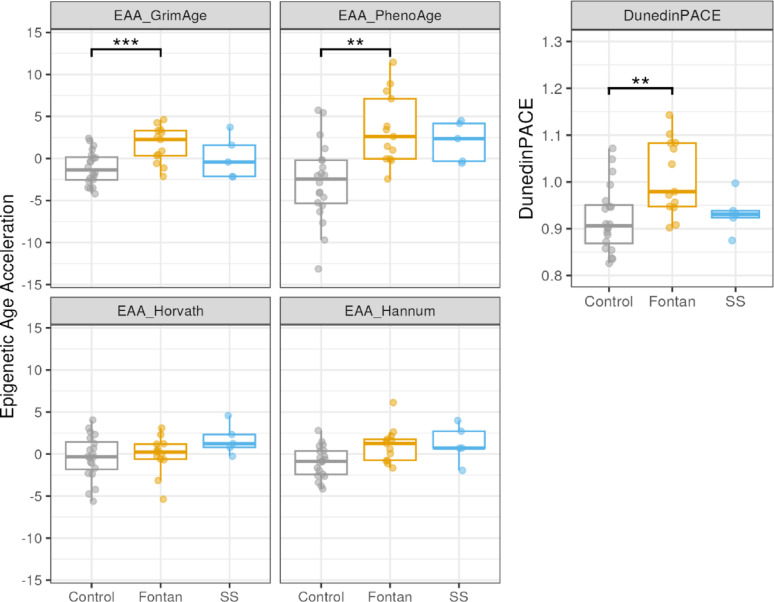
Fontan patients show higher EAA on GrimAge and PhenoAge, and DunedinPACE. EAA/DunedinPACE were compared between the Fontan, single surgery (SS) and control groups, and no significant differences among groups in EAAs of Horvath pan-tissue and Hannum (***: Bonferroni-adjusted *p* < 0.001, **: Bonferroni-adjusted *p* < 0.01)

To determine the difference in EAA between Fontan or SS groups and the controls, we applied the robust linear models as described in the Methods. The Fontan group had a significantly higher GrimAge EAA than the control group (t(27) = 4.78, Bonferroni-adjusted *p* < 0.001; Cohen’s f = 0.90). Similarly, the Fontan group’s PhenoAge EAA was significantly higher than controls (t(27) = 4.12, Bonferroni-adjusted *p* = 0.002; Cohen’s f = 0.82). Neither the Horvath nor the Hannum clock, showed significant differences between the Fontan group and controls, and no clocks showed significant differences between SS group and controls (Fig. 1 and Table 2).

**Table 2:** Test results for epigenetic age acceleration of first- and second-generation clocks, and DunedinPACE

Clock EAA	Comparison	β coefficient (SE)	β 95% CI	t(27)	*p*	Bonferroni-adjusted p	Cohen’s f	Contribution		
								IL-6 score	CRP score	DNAmPACKYRS
GrimAge	Fontan group and controls	4.211 (0.881)	2.484–5.939	4.778	7.4E-05	< 0.001	0.895	55.95%	44.25%	82.12%
PhenoAge		8.895 (2.158)	4.666–13.124	4.123	3.46E-04	0.002	0.818	53.20%	49.71%	62.10%
Dunedin PACE		0.136 (0.038)	0.062–0.21	3.593	1.67E-03	0.01	0.685	39.67%	54.0%	47.56%
Horvath		0.009 (1.287)	-2.513–2.531	0.007	9.94E-01	1	0.047			
Hannum		1.987 (0.975)	0.075–3.898	2.037	4.74E-02	0.284	0.441			
GrimAge	SS group and controls	1.951 (0.974)	0.042–3.861	2.003	4.93E-02	0.296	0.388			
PhenoAge		5.444 (2.385)	0.77–10.117	2.283	2.86E-02	0.171	0.483			
Dunedin PACE		0.042 (0.042)	-0.04–0.124	1.001	3.09E-01	1	0.153			
Horvath		2.215 (1.078)	0.102–4.328	2.055	6.09E-02	0.365	0.377			
Hannum		2.163 (1.422)	-0.624–4.95	1.521	1.35E-01	0.81	0.354			

For the five clocks, the Fontan and SS groups were compared to the control group. Contributions to EAA by DNAm-based IL-6, CRP scores and DNAmPACKYRS are shown for the clocks showing significant EAA differences

DunedinPACE is a metric estimating the rate of biological aging per chronological year and we found that the Fontan group had significantly higher DunedinPACE than controls (t(27) = 3.59, Bonferroni-adjusted *p* = 0.01; Cohen’s f = 0.69), while the SS group was not significantly different from controls (Fig. 1 and Table 2).

### Fontan group had lower estimated memory B cell and naïve CD4 + T cell proportions

As health conditions may relate to immune cell type proportions among individuals, we tested predicted cell type proportion differences among groups for each cell type. Estimated memory B cells (Bmem) (t(31) = 3.80, Bonferroni-adjusted *p* = 0.007; Cohen’s f = 0.60) and naïve CD4 + T cells (CD4nv) (t(31) = 7.01, Bonferroni-adjusted *p* < 0.001; Cohen’s f = 1.26) were significantly lower in the Fontan group than in controls. The remaining cell types did not show significant differences between the Fontan group and controls (Supplementary Figure S2 and Table S3), and there were no significant differences between the SS and control groups (Supplementary Figure S2 and Table S4).

### DNAm-based inflammation markers were higher in the Fontan group and contributed to GrimAge and PhenoAge EAA, and DunedinPACE

**Fig. 2:**
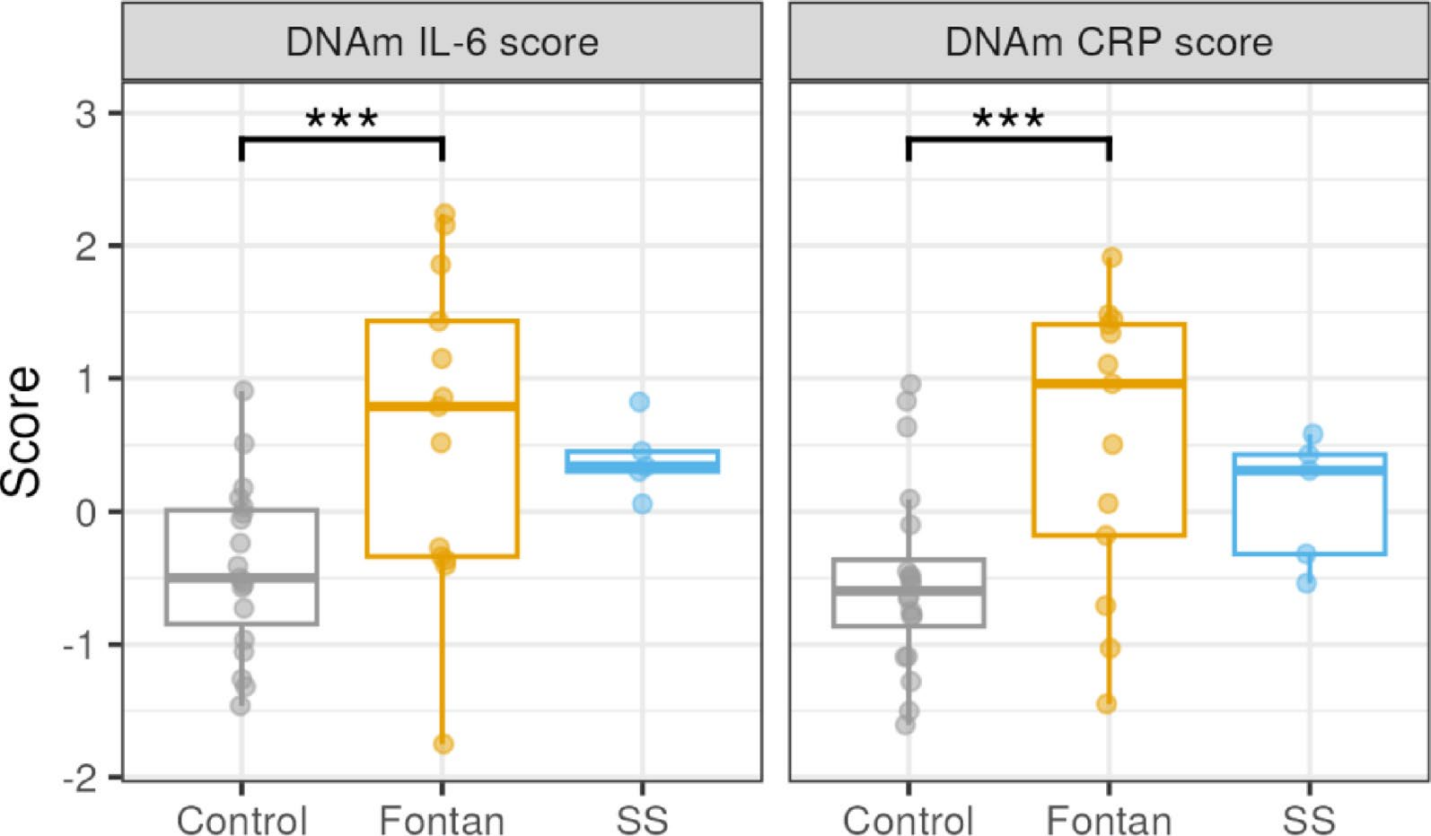
Fontan patients have higher DNAm-based IL-6 and CRP scores. DNAm-based IL-6 and CRP scores are compared between the Fontan, single surgery (ss) and control groups (***: Bonferroni-adjusted *p* < 0.001)

To further illuminate possible immune system alterations in our ACHD cohort, we used DNAm-based predictors to estimate inflammatory markers as surrogates for serum measures. DNAm-based IL-6 and CRP scores were normally distributed as tested by Shapiro–Wilk test (*W* = 0.95, *p* = 0.70 and *W* = 0.95, *p* = 0.12, respectively). Expectedly, a weak positive association was observed between predicted IL-6 and CRP scores (*r*(36) = 0.37, *p* = 0.02; Supplementary Figure S3). When comparing these predicted inflammatory profiles between groups, we observed that IL-6 and CRP scores of the Fontan group were significantly higher than controls (t(27) = 4.44, Bonferroni-adjusted *p* < 0.001 and t(27) = 4.69, Bonferroni-adjusted *p* < 0.001, respectively) with large Cohen’s effect size for IL-6 score (Cohen’s f = 0.84) and medium effect size for CRP score (Cohen’s f = 0.62) (Fig. 2 and Table S4). While IL-6 and CRP scores of the SS group were higher than controls, the trends did not reach statistical significance (t(27) = 2.27, Bonferroni-adjusted *p* = 0.054 and t(27) = 0.27, Bonferroni-adjusted *p* = 0.055, respectively) (Fig. 2 and Table S5).

Additionally, DNAmPACKYRS was moderately correlated with DNAm-based IL-6 and CRP scores (*r*(36) = 0.43, *p* = 0.007; *r*(36) = 0.42, *p* = 0.008, respectively) (Supplementary Figure S3). DNAmPACKYRS scores were significantly higher in Fontan patients compared to controls (t(27) = 4.19, Bonferroni-adjusted *p* = 0.001), with a large effect size (Cohen’s f = 0.92) (Supplementary Figure S4 and Table S5). No significant difference was observed between the SS group and controls.

Due to the observed inflammation score and differences between the groups and accelerated epigenetic age in some clocks, we sought to quantify the potential influence of DNAm-based predictors to the EAA and rate of aging differences between the Fontan group and controls. We calculated the contribution of IL-6, CRP scores, and DNAmPACKYRS separately to the GrimAge and PhenoAge EAA differences and to the DunedinPACE. In Fontan group GrimAge EAA, DNAm IL-6, CRP scores and DNAmPACKYRS contributed 55.95, 44.25, 82.12% respectively (Table 2). We observed similar contributions to the Fontan group PhenoAge EAA, with 53.20, 49.71, 62.10% respectively; and slightly lower IL-6 (39.67%), lower DNAmPACKYRS (47.56%) and higher CRP (54%) contributions to DunedinPACE (Table 2).

## Discussion

Adverse childhood experiences can become biologically embedded, resulting in disrupted physiology associated with poor long-term health outcomes, and widened gap between chronological and biological age [31, 53]. ACHD patients with Fontan circulation experience early childhood adversity of multiple surgical stresses, and have altered hemodynamics and physiology resulting in chronic morbidity. Using DNA methylation profiles from 18 ACHD patients and 20 controls in their young adulthood, we observed that ACHD patients with Fontan circulation had significantly accelerated biological aging, as measured by EAA, compared to healthy controls. Furthermore, our findings suggest that inflammation may contribute to increased EAA in the ACHD population, although further validation in larger, well-characterized cohorts is warranted.

Our study indicates that patients with Fontan circulation had significantly higher GrimAge and PhenoAge EAA, and DunedinPACE. All three of these measures of epigenetic aging had shown to be associated with cardiometabolic risk factors and mortality associated with cardiovascular disease [54, 55]. With improved survival, the ACHD population is at additional risk for acquired cardiovascular disease, and this higher morbidity/mortality risk of Fontan patients may potentially be indicated by the higher EAA as determined by the second-generation epigenetic clocks and rate-based clock. The EAA observed in this study may reflect a complex interplay between the underlying CHD condition, early-life surgical exposures, and the lifelong burden of comorbidities and chronic stress. However, our cross-sectional design precludes causal inference, and without longitudinal sampling beginning in the neonatal period or before surgery, it is not possible to determine whether EAA originated at birth, potentially due to underlying congenital factors, or accumulated over time. Future longitudinal studies starting in early life would be important to disentangle the temporal and mechanistic contribution to accelerated aging in ACHD.

The single surgery (SS) group showed a trend towards increased EAA but did not reach statistical significance. Although not significant, the SS group had lower or similar EAA, CRP, and IL-6 scores compared to the Fontan group but higher than controls. This suggested a potential cumulative effect of early life stress, from cardiac surgery and hospitalization, along with other adverse factors associated with ACHD, which may be reflected in DNA methylation changes observed in young adults and may be indicative of biological embedding.

Inflammaging, which describes the role of chronic, low grade, sterile inflammation in accelerated ageing, is increasingly recognized as an important factor in premature ageing [56]. We found that the DNAm-based proxies of inflammatory biomarkers CRP and IL-6 were significantly elevated in the Fontan subgroup of the ACHD population, consistent with measured IL-6 and CRP levels in patients with Fontan physiology [56, 57]. High sensitivity CRP measures have been shown to be elevated in ACHD and are associated with heart failure, arrhythmia and mortality [57], and the correlation of inflammation with positive EAA has been described in cardiovascular disease [58]. Predicted inflammation scores contributed to the EAA differences in GrimAge, PhenoAge and DunedinPACE, as these three clocks incorporated inflammatory biomarker for the clock training (PhenoAge: CRP, GrimAge: predicted B2M, DunedinPACE: CRP). The contribution analysis also indicated that these three clocks were sensitive to DNAm-predicted smoking scores. However, due to the lack of self-reported smoking data in our cohort, it remains unclear whether these DNAm smoking scores accurately reflected actual smoking behavior or rather capture a broader inflammatory state that may produce similar DNA methylation signatures, given that previous studies have reported associations between smoking and inflammation [59]. Considering the relatively low prevalence of smoking in the ACHD population previously reported (~ 10% current smokers and ~ 4% former smokers) [60], we hypothesize that the elevated DNAmPACKYRS observed in Fontan patients may, at least in part, reflect chronic inflammation rather than smoking exposure alone. Overall, our results may suggest inflammation is potentially an important contributor to EAA differences in this cohort. These predicted inflammatory biomarkers and their potential contribution to EAA offer insights into the functional status and chronic physiological stress state of this vulnerable population. These findings may suggest that longitudinal monitoring of inflammatory markers could be useful for evaluating disease progression and assessing treatment effects.

This study found that Fontan subgroup had significantly lower estimated memory B cell and naïve CD4 + T cells compared to healthy controls. These results, based on DNAm-predicted cell type proportions, may link to previous observation that early thymectomy, often part of surgical intervention in ACHD patients, had been associated with T-cell lymphopenia predominantly affecting memory and naïve cells [61]. Thymectomy is associated with long term autoimmune diseases, cancer, infections and atopic diseases [62] and may result in changes in the immunological system akin to aging [63]. However, these potential alterations in memory and naïve cell proportions require validation through direct measurement of cell counts.

Our study provides insight into accelerated epigenetic age in ACHD patients, especially in the high-risk Fontan subgroup, with some limitations. The small sample size, as indicated by the power analysis, was underpowered and may limit the generalizability of our findings. Therefore, these results require validation in independent cohorts with adequately powered sample sizes. We also acknowledge that in lieu of measured cell type proportions and inflammation markers, we used DNAm derived estimations, and the estimations have not yet been directly assessed in the participants. Even though these DNA methylation-based markers demonstrated high concordance with plasma protein measurements in generalized populations [37], our results should be validated using direct measurements in future studies. Additionally, the absence of detailed clinical profiling, such as comorbidities, anthropometric measures, smoking status, medication use, physical activity, and heart failure status, limited our ability to attribute the observed differences to specific underlying factors or to adjust for them in the statistical models. While the use of blood as a surrogate tissue may not fully capture tissue-specific aging processes, it can still provide valuable insights into systemic biological aging, particularly in ACHD patients who had been exposed to significant physiological stress from early-life surgeries and lifelong cardiovascular burden. Furthermore, due to the invasive nature of cardiac biopsies, especially in healthy controls, blood remains one of the most feasible options for assessing biological age in this study setting. Another limitation is the predominantly white, non-Hispanic population in the patient group, which may limit generalization of the results to other ancestries.

## Conclusions

ACHD patients with Fontan physiology had significant EAA as compared to their healthy counterparts. These epigenetic clocks can serve as a potential biomarker of the disease progression, which could be associated with chronic physiological stress resulting from immune and inflammation dysregulation. This study lays the groundwork for large-scale studies to investigate EAA, and the role of inflammation in EAA, in the broader ACHD population.

## Supplementary Information

Below is the link to the electronic supplementary material.

Supplementary Material 1

## Data Availability

DNA Methylation datasets available upon request with proper justification.

## References

[1] Van Der Linde D, Konings EEM, Slager MA, et al. Birth prevalence of congenital heart disease worldwide: a systematic review and meta-analysis. J Am Coll Cardiol. 2011. 10.1016/j.jacc.2011.08.025. 22078432 10.1016/j.jacc.2011.08.025

[2] Diller GP, Arvanitaki A, Opotowsky AR, et al. Lifespan perspective on congenital heart disease research: JACC state-of-the-art review. J Am Coll Cardiol. 2021;77(17):2219–35. 10.1016/J.JACC.2021.03.012. 33926659 10.1016/j.jacc.2021.03.012

[3] Gilboa SM, Devine OJ, Kucik JE, et al. Congenital Heart Defects in the United States: estimating the magnitude of the affected population in 2010. Circulation. 2016. 10.1161/CIRCULATIONAHA.115.019307. 27382105 10.1161/CIRCULATIONAHA.115.019307 PMC4942347

[4] Ntiloudi D, Giannakoulas G, Parcharidou D, Panagiotidis T, Gatzoulis MA, Karvounis H. Adult congenital heart disease: a paradigm of epidemiological change. Int J Cardiol. 2016. 10.1016/j.ijcard.2016.05.046. 27240150 10.1016/j.ijcard.2016.05.046

[5] Greutmann M, Tobler D, Kovacs AH, et al. Increasing mortality burden among adults with complex congenital heart disease. Congenit Heart Dis Published online. 2015. 10.1111/chd.12201. 10.1111/chd.12201 25043406

[6] Wienecke LM, Cohen S, Bauersachs J, Mebazaa A, Chousterman BG. Immunity and inflammation: the neglected key players in congenital heart disease? Heart Fail Rev. 2022;27(5):1957. 10.1007/S10741-021-10187-6. 34855062 10.1007/s10741-021-10187-6 PMC8636791

[7] Tournoy TK, Moons P, Daelman B, De Backer J. Biological age in congenital heart disease-exploring the ticking clock. J Cardiovasc Dev Dis. 2023. 10.3390/JCDD10120492. 38132660 10.3390/jcdd10120492 PMC10743752

[8] Constantine A, Ferrero P, Gribaudo E, et al. Morbidity and mortality in adults with a Fontan circulation beyond the fourth decade of life. Eur J Prev Cardiol. 2024;31(11):1316–23. 10.1093/EURJPC/ZWAE031. 38306409 10.1093/eurjpc/zwae031

[9] Iacobazzi D, Alvino VV, Caputo M, Madeddu P. Accelerated cardiac aging in patients with congenital heart disease. Front Cardiovasc Med. 2022;9:892861. 10.3389/FCVM.2022.892861. 35694664 10.3389/fcvm.2022.892861 PMC9177956

[10] Mattei AL, Bailly N, Meissner A. DNA methylation: a historical perspective. Trends Genet. 2022;38(7):676–707. 10.1016/J.TIG.2022.03.010/ASSET/36C89C3A-DA57-40BF-B492-179A108F171A/MAIN.ASSETS/GR3.JPG. 35504755 10.1016/j.tig.2022.03.010

[11] Salas LA, Zhang Z, Koestler DC, et al. Enhanced cell deconvolution of peripheral blood using DNA methylation for high-resolution immune profiling. Nat Commun. 2022. 10.1038/S41467-021-27864-7. 35140201 10.1038/s41467-021-27864-7 PMC8828780

[12] Houseman EA, Kile ML, Christiani DC, Ince TA, Kelsey KT, Marsit CJ. Reference-free deconvolution of DNA methylation data and mediation by cell composition effects. BMC Bioinformatics. 2016. 10.1186/S12859-016-1140-4. 27358049 10.1186/s12859-016-1140-4 PMC4928286

[13] Soriano-Tárraga C, Giralt-Steinhauer E, Mola-Caminal M, et al. Biological age is a predictor of mortality in ischemic stroke. Sci Rep. 2018. 10.1038/S41598-018-22579-0. 29515201 10.1038/s41598-018-22579-0 PMC5841388

[14] Roetker NS, Pankow JS, Bressler J, Morrison AC, Boerwinkle E. Prospective study of epigenetic age acceleration and incidence of cardiovascular disease outcomes in the ARIC study (Atherosclerosis Risk in Communities). Circ Genom Precis Med. 2018;11(3):e001937. 10.1161/CIRCGEN.117.001937. 29555670 10.1161/CIRCGEN.117.001937 PMC5863591

[15] Horvath S, Gurven M, Levine ME, et al. An epigenetic clock analysis of race/ethnicity, sex, and coronary heart disease. Genome Biol. 2016. 10.1186/s13059-016-1030-0. 27511193 10.1186/s13059-016-1030-0 PMC4980791

[16] Jiang R, Hauser ER, Kwee LC, et al. The association of accelerated epigenetic age with all-cause mortality in cardiac catheterization patients as mediated by vascular and cardiometabolic outcomes. Clin Epigenetics. 2022. 10.1186/S13148-022-01380-X. 36461124 10.1186/s13148-022-01380-x PMC9719253

[17] Nannini DR, Joyce BT, Zheng Y, et al. Epigenetic age acceleration and metabolic syndrome in the coronary artery risk development in young adults study. Clin Epigenetics. 2019. 10.1186/S13148-019-0767-1. 31730017 10.1186/s13148-019-0767-1 PMC6858654

[18] Stevenson AJ, Gadd DA, Hillary RF, et al. Creating and validating a DNA methylation-based proxy for interleukin-6. J Gerontol A Biol Sci Med Sci. 2021;76(12):2284–92. 10.1093/GERONA/GLAB046. 33595649 10.1093/gerona/glab046 PMC8599002

[19] Wielscher M, Mandaviya PR, Kuehnel B, et al. DNA methylation signature of chronic low-grade inflammation and its role in cardio-respiratory diseases. Nat Commun. 2022. 10.1038/S41467-022-29792-6. 35504910 10.1038/s41467-022-29792-6 PMC9065016

[20] Yousefi PD, Suderman M, Langdon R, Whitehurst O, Davey Smith G, Relton CL. DNA methylation-based predictors of health: applications and statistical considerations. Nat Rev Genet. 2022;23(6):369–83. 10.1038/s41576-022-00465-w. 35304597 10.1038/s41576-022-00465-w

[21] Oblak L, van der Zaag J, Higgins-Chen AT, Levine ME, Boks MP. A systematic review of biological, social and environmental factors associated with epigenetic clock acceleration. Ageing Res Rev. 2021. 10.1016/J.ARR.2021.101348. 33930583 10.1016/j.arr.2021.101348

[22] Jones MJ, Goodman SJ, Kobor MS. DNA methylation and healthy human aging. Aging Cell. 2015;14(6):924–32. 10.1111/ACEL.12349. 25913071 10.1111/acel.12349 PMC4693469

[23] Horvath S, Raj K. DNA methylation-based biomarkers and the epigenetic clock theory of ageing. Nat Rev Genet. 2018;19(6):371–84. 10.1038/S41576-018-0004-3. 29643443 10.1038/s41576-018-0004-3

[24] McEwen LM, Jones MJ, Lin DTS, et al. Systematic evaluation of DNA methylation age estimation with common preprocessing methods and the Infinium MethylationEPIC BeadChip array. Clin Epigenetics. 2018. 10.1186/S13148-018-0556-2. 30326963 10.1186/s13148-018-0556-2 PMC6192219

[25] Horvath S. DNA methylation age of human tissues and cell types. Genome Biol. 2013. 10.1186/GB-2013-14-10-R115. 24138928 10.1186/gb-2013-14-10-r115 PMC4015143

[26] Wang C, Ni W, Yao Y, et al. DNA methylation-based biomarkers of age acceleration and all-cause death, myocardial infarction, stroke, and cancer in two cohorts: the NAS, and KORA F4. EBioMedicine. 2021. 10.1016/J.EBIOM.2020.103151. 33279859 10.1016/j.ebiom.2020.103151 PMC7724153

[27] Perna L, Zhang Y, Mons U, Holleczek B, Saum KU, Brenner H. Epigenetic age acceleration predicts cancer, cardiovascular, and all-cause mortality in a German case cohort. Clin Epigenetics. 2016. 10.1186/S13148-016-0228-Z. 27274774 10.1186/s13148-016-0228-z PMC4891876

[28] Soriano-Tárraga C, Giralt-Steinhauer E, Mola-Caminal M, et al. Ischemic stroke patients are biologically older than their chronological age. Aging. 2016;8(11):2655–66. 10.18632/AGING.101028. 27922817 10.18632/aging.101028 PMC5191861

[29] Horvath S, Erhart W, Brosch M, et al. Obesity accelerates epigenetic aging of human liver. Proc Natl Acad Sci U S A. 2014;111(43):15538–43. 10.1073/PNAS.1412759111. 25313081 10.1073/pnas.1412759111 PMC4217403

[30] Aristizabal MJ, Anreiter I, Halldorsdottir T, et al. Biological embedding of experience: a primer on epigenetics. Proc Natl Acad Sci U S A. 2020;117(38):23261–9. 10.1073/pnas.1820838116. 31624126 10.1073/pnas.1820838116 PMC7519272

[31] McCrory C, Fiorito G, O’Halloran AM, Polidoro S, Vineis P, Kenny RA. Early life adversity and age acceleration at mid-life and older ages indexed using the next-generation GrimAge and Pace of Aging epigenetic clocks. Psychoneuroendocrinology. 2022;137:105643. 10.1016/J.PSYNEUEN.2021.105643. 34999481 10.1016/j.psyneuen.2021.105643

[32] Desiderio A, Pastorino M, Campitelli M, et al. DNA methylation in cardiovascular disease and heart failure: novel prediction models? Clin Epigenetics. 2024. 10.1186/S13148-024-01722-X. 39175069 10.1186/s13148-024-01722-x PMC11342679

[33] Zhang F, Deng S, Zhang J, et al. Causality between heart failure and epigenetic age: a bidirectional Mendelian randomization study. ESC Heart Fail. 2023;10(5):2903–13. 10.1002/EHF2.14446. 37452462 10.1002/ehf2.14446 PMC10567637

[34] Illumina. Convert DNA 1. Follow the Instructions in the Zymo EZ DNA Infinium HD Methylation Assay Manual Workflow Checklist; 2020.

[35] Merrill SM, Gladish N, Fu MP, et al. Associations of peripheral blood DNA methylation and estimated monocyte proportion differences during infancy with toddler attachment style. Attach Hum Dev. 2023;25(1):132–61. 10.1080/14616734.2021.1938872. 34196256 10.1080/14616734.2021.1938872

[36] Konwar C, Asiimwe R, Inkster AM, et al. Risk-focused differences in molecular processes implicated in SARS-CoV-2 infection: corollaries in DNA methylation and gene expression. Epigenetics Chromatin. 2021. 10.1186/S13072-021-00428-1. 34895312 10.1186/s13072-021-00428-1 PMC8665859

[37] Murat K, Grüning B, Poterlowicz PW, Westgate G, Tobin DJ, Poterlowicz K. Ewastools: Infinium human methylation beadchip pipeline for population epigenetics integrated into Galaxy. Gigascience. 2020. 10.1093/GIGASCIENCE/GIAA049. 32401319 10.1093/gigascience/giaa049 PMC7219210

[38] Aryee MJ, Jaffe AE, Corrada-Bravo H, et al. Minfi: a flexible and comprehensive Bioconductor package for the analysis of Infinium DNA methylation microarrays. Bioinformatics. 2014;30(10):1363–9. 10.1093/BIOINFORMATICS/BTU049. 24478339 10.1093/bioinformatics/btu049 PMC4016708

[39] Pidsley R, Y Wong CC, Volta M, Lunnon K, Mill J, Schalkwyk LC. A data-driven approach to preprocessing Illumina 450K methylation array data. BMC Genomics. 2013;14(1):293. 10.1186/1471-2164-14-293. 23631413 10.1186/1471-2164-14-293 PMC3769145

[40] Teschendorff AE, Marabita F, Lechner M, et al. A beta-mixture quantile normalization method for correcting probe design bias in Illumina Infinium 450 k DNA methylation data. Bioinformatics. 2013;29(2):189–96. 10.1093/BIOINFORMATICS/BTS680. 23175756 10.1093/bioinformatics/bts680 PMC3546795

[41] Leek JT, Johnson WE, Parker HS, Jaffe AE, Storey JD. The sva package for removing batch effects and other unwanted variation in high-throughput experiments. Bioinformatics. 2012;28(6):882–3. 10.1093/BIOINFORMATICS/BTS034. 22257669 10.1093/bioinformatics/bts034 PMC3307112

[42] Engelbrecht HR, Merrill SM, Gladish N, et al. Sex differences in epigenetic age in Mediterranean high longevity regions. Frontiers in Aging. 2022;3:1007098. 10.3389/FRAGI.2022.1007098/BIBTEX. 36506464 10.3389/fragi.2022.1007098 PMC9726738

[43] Zheng SC, Beck S, Jaffe AE, et al. Correcting for cell-type heterogeneity in epigenome-wide association studies: revisiting previous analyses. Nat Methods. 2017;14(3):216–7. 10.1038/NMETH.4187. 28245219 10.1038/nmeth.4187 PMC6659733

[44] Jones MJ, Moore SR, Kobor MS. Principles and challenges of applying epigenetic epidemiology to psychology. Annu Rev Psychol. 2018;69:459–85. 10.1146/ANNUREV-PSYCH-122414-033653. 29035689 10.1146/annurev-psych-122414-033653

[45] Filzmoser P, Hron K, Reimann C. Principal component analysis for compositional data with outliers. Environmetrics. 2009;20(6):621–32. 10.1002/ENV.966.

[46] Hannum G, Guinney J, Zhao L, et al. Genome-wide methylation profiles reveal quantitative views of human aging rates. Mol Cell. 2013;49(2):359–67. 10.1016/J.MOLCEL.2012.10.016. 23177740 10.1016/j.molcel.2012.10.016 PMC3780611

[47] Lu AT, Quach A, Wilson JG, et al. DNA methylation GrimAge strongly predicts lifespan and healthspan. Aging. 2019;11(2):303–27. 10.18632/AGING.101684. 30669119 10.18632/aging.101684 PMC6366976

[48] Levine ME, Lu AT, Quach A, et al. An epigenetic biomarker of aging for lifespan and healthspan. Aging. 2018;10(4):573–91. 10.18632/AGING.101414. 29676998 10.18632/aging.101414 PMC5940111

[49] Belsky DW, Caspi A, Corcoran DL, et al. DunedinPACE, a DNA methylation biomarker of the pace of aging. Elife. 2022. 10.7554/ELIFE.73420. 35029144 10.7554/eLife.73420 PMC8853656

[50] Torchiano M. Efficient Effect Size Computation [R package effsize version 0.8.1]. *CRAN: Contributed Packages*. Published online October 5, 2020. 10.32614/CRAN.PACKAGE.EFFSIZE

[51] Merrill SM, Moore SR, Gladish N, et al. Paternal adverse childhood experiences: associations with infant DNA methylation. Dev Psychobiol. 2021. 10.1002/DEV.22174. 34333774 10.1002/dev.22174

[52] Roberts AL, Gladish N, Gatev E, et al. Exposure to childhood abuse is associated with human sperm DNA methylation. Transl Psychiatry. 2018. 10.1038/S41398-018-0252-1. 30279435 10.1038/s41398-018-0252-1 PMC6168447

[53] Berens AE, Jensen SKG, Nelson CA. Biological embedding of childhood adversity: from physiological mechanisms to clinical implications. BMC Med. 2017;15(1):1–12. 10.1186/S12916-017-0895-4/PEER-REVIEW. 28724431 10.1186/s12916-017-0895-4 PMC5518144

[54] Ma Q, Li BL, Yang L, et al. Association between phenotypic age and mortality in patients with multivessel coronary artery disease. Dis Markers. 2022. 10.1155/2022/4524032. 35069932 10.1155/2022/4524032 PMC8776473

[55] Ammous F, Zhao W, Ratliff SM, et al. Epigenetic age acceleration is associated with cardiometabolic risk factors and clinical cardiovascular disease risk scores in African Americans. Clin Epigenetics. 2021. 10.1186/S13148-021-01035-3. 33726838 10.1186/s13148-021-01035-3 PMC7962278

[56] Franceschi C, Garagnani P, Parini P, Giuliani C, Santoro A. Inflammaging: a new immune-metabolic viewpoint for age-related diseases. Nat Rev Endocrinol. 2018;14(10):576–90. 10.1038/S41574-018-0059-4. 30046148 10.1038/s41574-018-0059-4

[57] Geenen LW, Baggen VJM, Van Den Bosch AE, et al. Prognostic value of C-reactive protein in adults with congenital heart disease. Heart. 2020;107(6):474–81. 10.1136/HEARTJNL-2020-316813. 33060260 10.1136/heartjnl-2020-316813 PMC7925816

[58] Liu D, Aziz NA, Pehlivan G, Breteler MMB. Cardiovascular correlates of epigenetic aging across the adult lifespan: a population-based study. Geroscience. 2023;45(3):1605–18. 10.1007/S11357-022-00714-0. 36752898 10.1007/s11357-022-00714-0 PMC10400487

[59] Zhang W, Lin H, Zou M, et al. Nicotine in inflammatory diseases: anti-inflammatory and pro-inflammatory effects. Front Immunol Front Media SA. 2022. 10.3389/fimmu.2022.826889. 10.3389/fimmu.2022.826889 PMC8895249 35251010

[60] Engelfriet PM, Drenthen W, Pieper PG, et al. Smoking and its effects on mortality in adults with congenital heart disease. Int J Cardiol. 2008;127(1):93–7. 10.1016/j.ijcard.2007.05.008. 17692954 10.1016/j.ijcard.2007.05.008

[61] Gudmundsdottir J, Lundqvist C, Ijspeert H, et al. T-cell receptor sequencing reveals decreased diversity 18 years after early thymectomy. J Allergy Clin Immunol. 2017;140(6):1743-1746.e7. 10.1016/J.JACI.2017.08.002. 28866385 10.1016/j.jaci.2017.08.002

[62] Gudmundsdottir J, Söderling J, Berggren H, et al. Long-term clinical effects of early thymectomy: associations with autoimmune diseases, cancer, infections, and atopic diseases. J Allergy Clin Immunol. 2018;141(6):2294-2297.e8. 10.1016/j.jaci.2018.01.037. 29454003 10.1016/j.jaci.2018.01.037

[63] Goronzy JJ, Fang F, Cavanagh MM, Qi Q, Weyand CM. Naive T cell maintenance and function in human aging. J Immunol. 2015;194(9):4073–80. 10.4049/JIMMUNOL.1500046. 25888703 10.4049/jimmunol.1500046 PMC4452284

