# Supplementary material for: Epigenetic age acceleration in young adults with congenital heart disease
Source: Clin Epigenetics. 2026 Feb 15;18:87. doi: 10.1186/s13148-026-02049-5 (PMC13202995; doi:10.1186/s13148-026-02049-5)
Supplement: Supplementary file 1 — Supplementary Material 1 [file 13148_2026_2049_MOESM1_ESM.docx]

# Supplementary figures


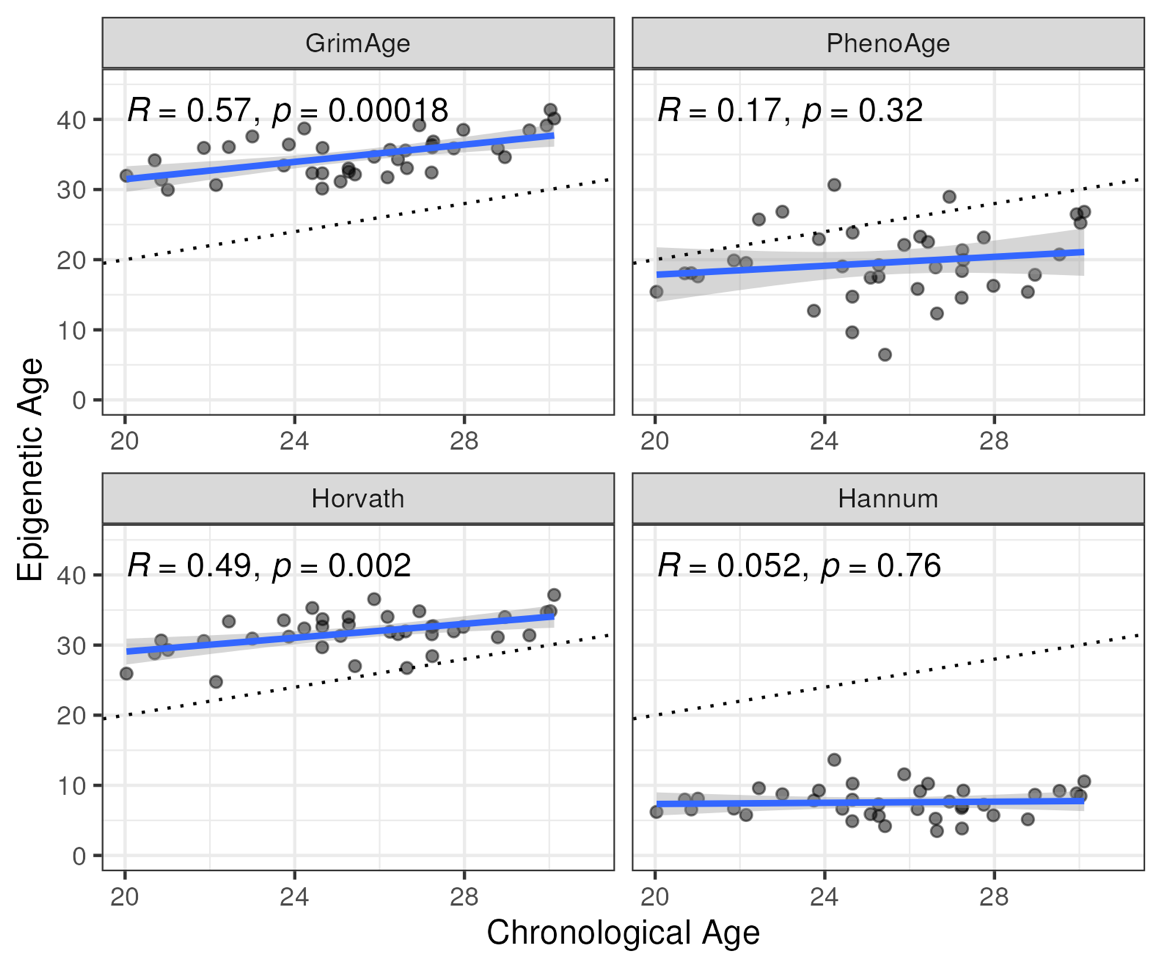


Figure S1. Scatter plot of epigenetic age estimates and chronological age for all samples (n=38). Pearson's *r* and *p* are reported for each epigenetic clock with dotted line indicating x=y.


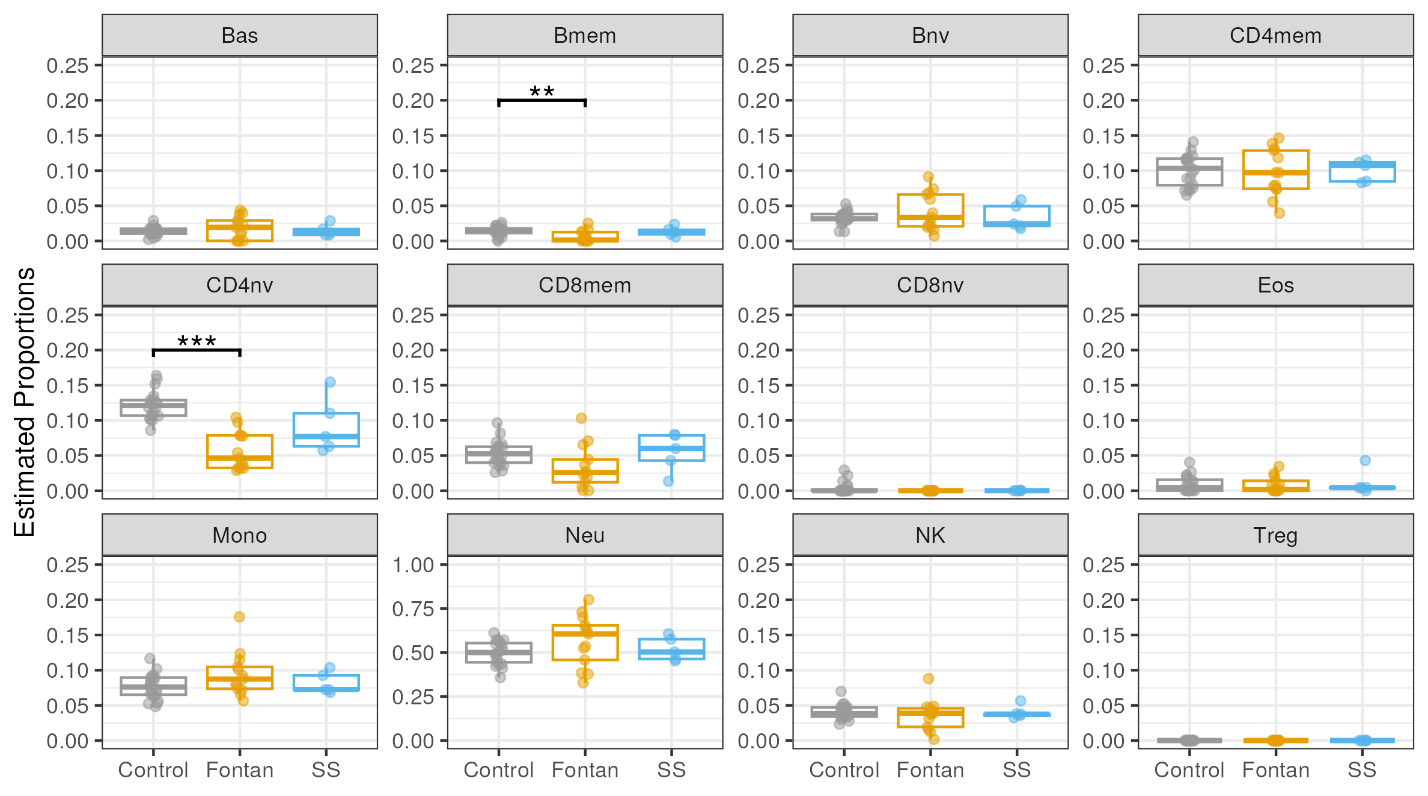


Figure S2. Differences in predicted cell type proportions of basophils (Bas), memory B cells (Bmem), naïve B cells(Bnv), memory CD4 + T cells (CD4mem), naïve CD4 + T cells (CD4nv), memory CD8 + T cells (CD8mem), naïve CD8 + T cells (CD8nv), eosinophils (Eos), monocytes (Mono), neutrophils (Neu), natural killer (NK), and T regulatory cells (Treg) among the three groups. ***: Bonferroni-adjusted *p* <. 0001, **: Bonferroni-adjusted *p* <.01


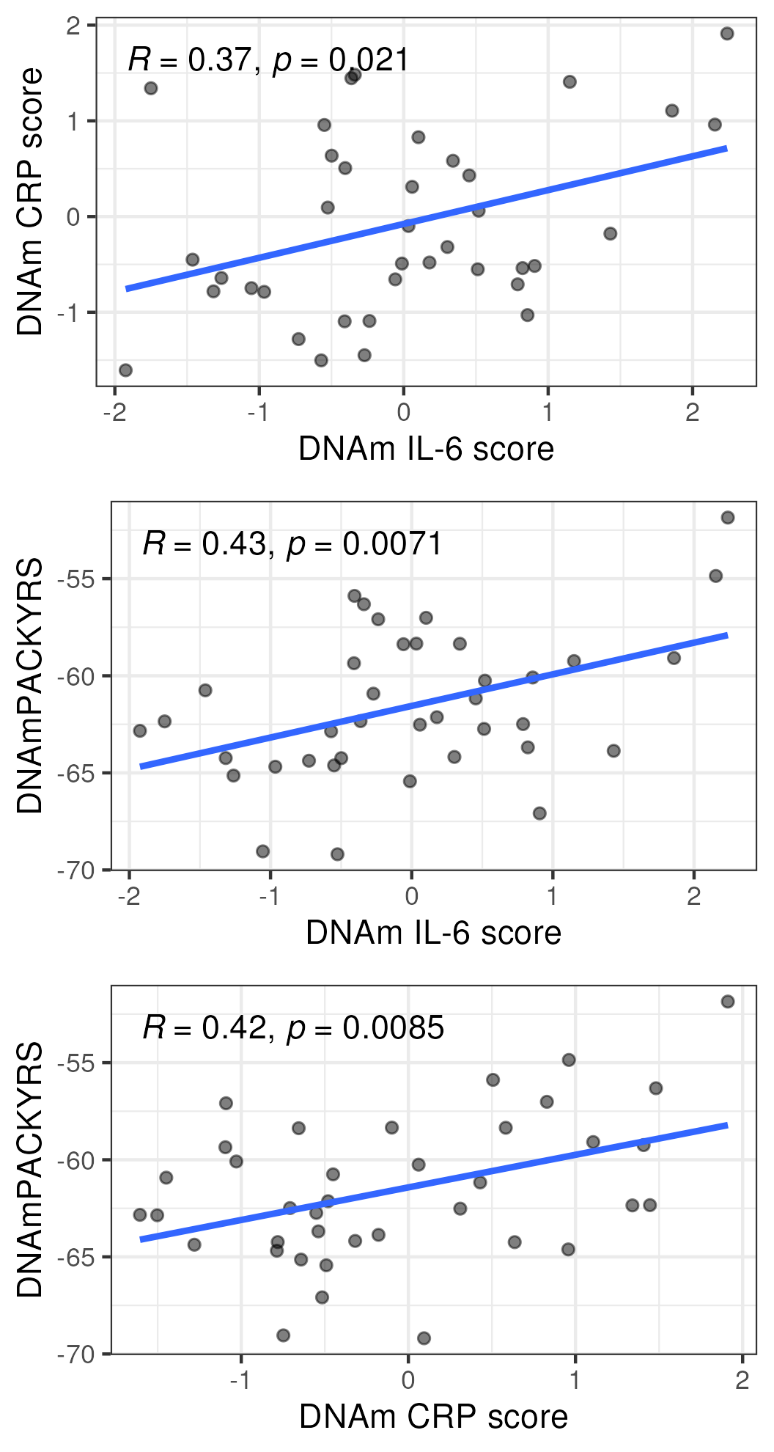


Figure S3. Scatter plot of DNAm-based IL-6 score, CRP score and DNAmPACKYRS showing positive correlation (n=38).


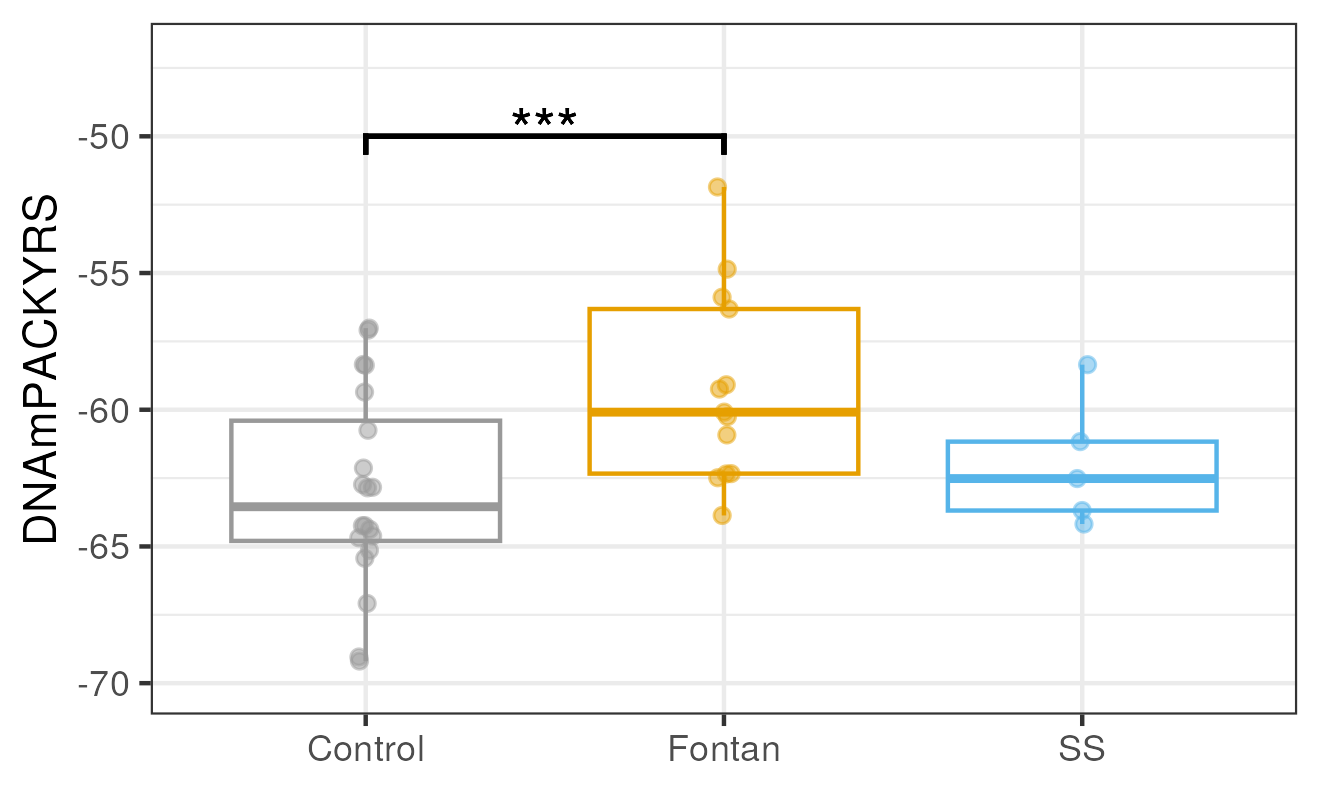


Figure S4. Comparison of DNAmPACKYRS among the Fontan, single surgery (ss) and control groups (***: Bonferroni-adjusted p < 0.001).

# Supplementary tables

Table S1. Details of cardiac anatomy and surgical interventions.

| **Fontan** | **Anatomy** | **Surgical interventions (age at intervention)** |
| --- | --- | --- |
| 001F | Hypoplastic Left Heart Syndrome (double outlet right ventricle, mitral atresia, side-by-side great arteries) with transverse arch hypoplasia, coarctation of the aorta and Patrent Ductus Arteriosus | Balloon atrial septostomy (1 day old) |
|  |  | Aortic Arch advancement, Pulmonary Artery band, atrial septectomy (1 month) |
|  |  | Bidirectional Glenn (9 month) |
|  |  | Fenestrated lateral tunnel Fontan, incision of pulmonary venous confluence (4.5 years) |
| 002F | Tricuspid atresia and d-TGA, VSD, hypoplasia of the aortic arch | Atrial septostomy (1 week) |
|  |  | Pulmonary artery banding (1 month) |
|  |  | Extracardiac fenestrated Fontan procedure (2 years) |
| 003F | Heterotaxy syndrome - asplenia type with unbalanced atrioventricular septal defect, pulmonary atresia,L-Transposition of great arteries | Bilateral BTT shunt (1 month) |
|  |  | Glenn and fenestrated Fontan procedure (10 years) |
| 004F | Dextrocardia with d-looped ventricles (right sided hypoplastic RV and tricuspid valve), large VSD, L-transposition of the great vessels (aorta anterior and leftward), valvar and subvalvar pulmonary stenosis. | Bidirectional Glenn and main pulmonary artery oversewing (1 month) |
|  |  | balloon atrial septostomy for chylous effusions (1 month) |
|  |  | RPA banding to recreate classic Glenn physiology (1 month) |
|  |  | Lateral tunnel Fontan completion (tiny fenestration) and MPA-RPA patch reconstruction (3 years) |
| 005F | Tricuspid and pulmonary atresia with severely hypoplastic right ventricle. | BTT shunt (2 months of age) |
|  |  | Bidirectional Glenn (at 1 year) |
|  |  | Extra cardiac conduit Fontan (7 years of age) |
| 006F | Tricuspid atresia | Balloon atrial septostomy (2 days) |
|  |  | Right modified BTT shunt with 4mm Gortex conduit (1 month) |
|  |  | Bidirectional Glenn, shunt take down (9 months) |
|  |  | Lateral tunnel Fontan with 3mm fenestration (4 years of age) |
| 007F | Tricuspid atresia | Balloon atrial septostomy (neonate) |
|  |  | Left BTT shunt (1 month) |
|  |  | Bidirectional Glenn (10 months) |
|  |  | Fontan with fenestration (2 years) |
| 008F | Heterotaxy syndrome (right isomerism type) with an double inlet left ventricle, multiple ventricular septal defects, pulmonary atresia, and bilateral superior venae cava. | BTT shunt (1 month) |
|  |  | Bilateral Glenn anastomosis (infant) |
|  |  | Lateral tunnel fenestrated Fontan (5 years) |
| 009F | Double inlet left ventricle, L-transposition of the great arteries | Damus-Kaye-Stansel procedure with right sided BTT shunt (1 month) |
|  |  | Glenn anastomosis (1 year of age) |
|  |  | Fenestrated Fontan (5 years of age) |
| 010F | Hypoplastic left heart syndrome | Norwood operation with 4 mm Gortex Blalock-Taussig shunt (1 month) |
|  |  | Bidirectional Glenn operation with RPA augmentation with pericardial patch and BT shunt takedown (6 months of age) |
|  |  | Lateral tunnel fenestrated Fontan (3 years of age) |
| 011F | Hypoplastic left heart syndrome (MA/AS) | Norwood procedure with right sided modified BTT shunt (1 week of age) |
|  |  | Bidirectional Glenn, BT shunt takedown (8 months of age) |
|  |  | Lateral tunnel fenestrated Fontan with delayed sternal closure for wound infection/mediastinitis (2 years of age) |
| 012F | Double-inlet left ventricle with d-transposition of the great arteries (d-looped ventricles), hypoplastic right ventricle, subaortic stenosis, and coarctation of the aorta. | Arch advancement, pulmonary artery banding, and PDA ligation (2 days of age) |
|  |  | Bilateral bidirectional Glenn, MPA debanding and oversewing, subaortic resection, and atrial septectomy (4 months of age) |
|  |  | 4 mm fenestrated lateral tunnel Fontan completion, subaortic resection, Dacron patch repair of subaortic ventricular aneurysm, bilateral PA patch augmentation (15 months of age) |
|  |  | Damus-Kaye-Stansel operation for recurrent subaortic stenosis (3 years of age) |
| 013F | Heterotaxy syndrome - asplenia type / right isomerism; abdominal; stomach; dextrocardia, right sided IVC, bilateral SVCs, atrial situs inversus, secundum and primum ASD, common AV valve, single ventricle, Double outlet right ventricle with pulmonary stenosis, aorta anterior with right arch and mirror imaged branching. | Pulmonary balloon valvuloplasty (1 week old) |
|  |  | Bilateral bidirectional Glenn procedure (1 year old) |
|  |  | Fenestrated, extracardiac Fontan circulation completion (3 years old) |
| **Single Surgery** | **Anatomy** | **Surgical Procedure (age at surgery)** |
| SS1 | Transposition of Great Arteries with intact ventricular septum | Arterial Switch Operation (1week) |
| SS2 | Perimembranous Ventricular septal defect (VSD), Atrial Septal Defect (ASD), Patent Ductus Arteriosus (PDA) | Closure of VSD, ASD and PDA ligation (2 months of age) |
| SS3 | Tetralogy of Fallot wth pulmonary stenosis | tetralogy of Fallot repair with Transannular patch repair, VSD closure (4 months of age) |
| SS4 | Tetralogy of Fallot with pulmonary stenosis | tetralogy of Fallot repair with Transannular patch repair, VSD closure (11 months of age) |
| SS5 | Tetralogy of Fallot with pulmonary stenosis | tetralogy of Fallot repair with Transannular patch repair, VSD closure (9 months of age) |

Table S2. Pearson correlations and mean absolute errors of epigenetic clock estimates with chronological age.

| **clock** | ***r*** | **MAE** | **MaxAE** |
| --- | --- | --- | --- |
| GrimAge | 0.572 | 9.35 | 14.57 |
| PhenoAge | 0.167 | 6.73 | 18.97 |
| Horvath | 0.487 | 6.27 | 10.92 |
| Hannum | 0.052 | 17.96 | 23.64 |

r: Pearson correlation between epigenetic age and chronological age. MAE: mean absolute error in years. MaxAE: maximum absolute error in years

Table S3. Test statistics of immune cell type proportion comparisons between Fontan and controls (baseline).

| Cell Type | *β* coefficient (SE) | *β* 95% CI | t(31) | *p* | Bonferroni-adjusted *p* | Cohen's f |
| --- | --- | --- | --- | --- | --- | --- |
| Bas | 1.62E-03(3.97E-03) | -6.17E-03 - 9.4E-03 | 0.408 | 6.89E-01 | 1 | 0.148 |
| Bmem | -1.11E-02(2.93E-03) | -1.68E-02 - -5.36E-03 | -3.792 | 6.47E-04 | 0.007 | 0.601 |
| Bnv | 6.78E-03(5.8E-03) | -4.6E-03 - 1.82E-02 | 1.168 | 2.64E-01 | 1 | 0.285 |
| CD4mem | -4.52E-03(8.02E-03) | -2.03E-02 - 1.12E-02 | -0.564 | 5.85E-01 | 1 | 0.12 |
| CD4nv | -6.57E-02(9.38E-03) | -8.41E-02 - -4.73E-02 | -7.008 | 5.94E-08 | <0.001 | 1.256 |
| CD8mem | -1.83E-02(9.36E-03) | -3.66E-02 - 6.66E-05 | -1.953 | 6.18E-02 | 0.679 | 0.331 |
| CD8nv | -6.13E-07(5.11E-07) | -1.61E-06 - 3.89E-07 | -1.2 | 2.16E-01 | 1 | 0.25 |
| Eos | -1.48E-03(3.84E-03) | -9E-03 - 6.05E-03 | -0.384 | 7.04E-01 | 1 | 0.003 |
| Mono | 7.61E-03(8.05E-03) | -8.17E-03 - 2.34E-02 | 0.945 | 3.59E-01 | 1 | 0.249 |
| Neu | 8.19E-02(4.01E-02) | 3.36E-03 - 1.6E-01 | 2.044 | 5.29E-02 | 0.582 | 0.399 |
| NK | -8.86E-03(5.09E-03) | -1.88E-02 - 1.11E-03 | -1.742 | 9.45E-02 | 1 | 0.286 |

basophils (Bas), memory B cells (Bmem), naïve B cells(Bnv), memory CD4 + T cells (CD4mem), naïve CD4 + T cells (CD4nv), memory CD8 + T cells (CD8mem), naïve CD8 + T cells (CD8nv), eosinophils (Eos), monocytes (Mono), neutrophils (Neu), natural killer (NK)

Table S4. Test statistics of immune cell type proportions comparison between SS and controls (baseline).

| Cell Type | β coefficient (SE) | β 95% CI | t(31) | p | Bonferroni-adjusted p | Cohen's f |
| --- | --- | --- | --- | --- | --- | --- |
| Bas | 1.1E-03(5.32E-03) | -9.33E-03 - 1.15E-02 | 0.206 | 8.38E-01 | 1 | 0.057 |
| Bmem | -2.07E-03(3.92E-03) | -9.75E-03 - 5.61E-03 | -0.528 | 6.04E-01 | 1 | 0.056 |
| Bnv | 3.86E-03(7.78E-03) | -1.14E-02 - 1.91E-02 | 0.496 | 6.18E-01 | 1 | 0.122 |
| CD4mem | 4.48E-03(1.08E-02) | -1.66E-02 - 2.56E-02 | 0.417 | 6.75E-01 | 1 | 0.048 |
| CD4nv | -3.66E-02(1.26E-02) | -6.12E-02 - -1.19E-02 | -2.909 | 9.45E-03 | 0.104 | 0.383 |
| CD8mem | 9.22E-03(1.25E-02) | -1.54E-02 - 3.38E-02 | 0.735 | 4.67E-01 | 1 | 0.122 |
| CD8nv | -2.22E-07(6.85E-07) | -1.56E-06 - 1.12E-06 | -0.323 | 7.56E-01 | 1 | 0.192 |
| Eos | -5.01E-04(5.14E-03) | -1.06E-02 - 9.58E-03 | -0.097 | 9.25E-01 | 1 | 0.121 |
| Mono | 3.24E-03(1.08E-02) | -1.79E-02 - 2.44E-02 | 0.3 | 7.62E-01 | 1 | 0.03 |
| Neu | 3.94E-03(5.37E-02) | -1.01E-01 - 1.09E-01 | 0.073 | 9.41E-01 | 1 | 0.024 |
| NK | 7.34E-04(6.82E-03) | -1.26E-02 - 1.41E-02 | 0.108 | 9.14E-01 | 1 | 0.028 |

basophils (Bas), memory B cells (Bmem), naïve B cells(Bnv), memory CD4 + T cells (CD4mem), naïve CD4 + T cells (CD4nv), memory CD8 + T cells (CD8mem), naïve CD8 + T cells (CD8nv), eosinophils (Eos), monocytes (Mono), neutrophils (Neu), natural killer (NK).

Table S5. Test results for DNAm-based inflammation scores and DNAmPACKYRS for Fontan and SS groups as compared to controls.

| DNAm score | Comparison | β coefficient (SE) | β 95% CI | t(27) | p | Bonferroni-adjusted p | Cohen's f |
| --- | --- | --- | --- | --- | --- | --- | --- |
| IL-6 | Fontan | 1.679(0.378) | 0.937 - 2.42 | 4.435 | 1.82E-04 | <0.001 | 0.835 |
| CRP | Fontan | 1.853(0.395) | 1.079 - 2.627 | 4.691 | 1.23E-04 | <0.001 | 0.617 |
| DNAmPACKYRS | Fontan | 7.095(1.695) | 3.774 - 10.417 | 4.187 | 2.69E-04 | 0.001 | 0.924 |
| IL-6 | SS | 0.95(0.418) | 0.131 - 1.77 | 2.272 | 2.72E-02 | 0.054 | 0.448 |
| CRP | SS | 0.991(0.437) | 0.136 - 1.847 | 2.271 | 2.73E-02 | 0.055 | 0.327 |
| DNAmPACKYRS | SS | 2.623(1.873) | -1.048 - 6.294 | 1.401 | 1.65E-01 | 0.331 | 0.339 |
